# Rapid Discovery and Detection of *Haemaphysalis longicornis* through the Use of Passive Surveillance and Collaboration: Building a State Tick-Surveillance Network

**DOI:** 10.3390/ijerph18157980

**Published:** 2021-07-28

**Authors:** Rebecca T. Trout Fryxell, Dené N. Vann, Rebecca A. Butler, Dave J. Paulsen, Jennifer G. Chandler, Micah P. Willis, Heidi M. Wyrosdick, John J. Schaefer, Richard W. Gerhold, Daniel M. Grove, Jennie Z. Ivey, Kevin W. Thompson, Roger D. Applegate, Joy Sweaney, Sterling Daniels, Samantha Beaty, Douglas Balthaser, James D. Freye, James W. Mertins, Denise L. Bonilla, Kevin Lahmers

**Affiliations:** 1 Department of Entomology and Plant Pathology, University of Tennessee, Knoxville, TN 37996, USA; rbutle25@vols.utk.edu (R.A.B.); dpaulsen@utk.edu (D.J.P.); jchand11@utk.edu (J.G.C.); 2 Department of Biomedical Diagnostic Sciences, College of Veterinary Medicine, University of Tennessee, Knoxville, TN 37996, USA; dvann4@vols.utk.edu (D.N.V.); hwyrosdi@utk.edu (H.M.W.); jschaef6@utk.edu (J.J.S.); rgerhold@utk.edu (R.W.G.); 3 Department of Agricultural Leadership, Education and Communications, University of Tennessee, Knoxville, TN 37996, USA; mwill187@vols.utk.edu; 4 Department of Forestry, Wildlife and Fisheries, University of Tennessee, Knoxville, TN 37996, USA; dgrove@utk.edu; 5 Department of Animal Science, University of Tennessee, Knoxville, TN 37996, USA; jzivey@utk.edu; 6 Middle Tennessee Research and Education Center, University of Tennessee, Knoxville, TN 37996, USA; kthomp44@utk.edu; 7 Wildlife and Forestry Division, Tennessee Wildlife Resources Agency, Nashville, TN 37211, USA; roger.applegate@tn.gov (R.D.A.); joy.sweaney@tn.gov (J.S.); sterling.daniels@tn.gov (S.D.); 8 State Veterinarians’ Office, Ellington Agricultural Center, Tennessee Department of Agriculture, Nashville, TN 37220, USA; samantha.beaty@tn.gov (S.B.); douglas.balthaser@tn.gov (D.B.); 9 Tennessee Veterinary Services, Animal and Plant Health Inspection Service, United States Department of Agriculture, Madison, TN 37220, USA; james.d.freye@aphis.usda.gov; 10 National Veterinary Services Laboratories, Veterinary Services, Animal and Plant Health Inspection Service, U.S. Department of Agriculture, Ames, IA 50010, USA; james.w.mertins@usda.gov; 11 Veterinary Services, Animal and Plant Health Inspection Service, U.S. Department of Agriculture, Fort Collins, CO 80521, USA; denise.l.bonilla@usda.gov; 12 Virginia Tech Animal Laboratory Services and Department of Biomedical Sciences and Pathobiology, VA Polytechnic Institute and State University, Blacksburg, VA 24061, USA; klahmers@vt.edu

**Keywords:** tick, distribution zoonoses, collaboration, detection, OneHealth

## Abstract

Between March 2019 and February 2020, Asian long-horned ticks (*Haemaphysalis longicornis* Neumann, 1901) were discovered and collected for the first time in one middle and seven eastern Tennessee counties, facilitated by a newly developed passive and collaborative tick-surveillance network. Network collaborators included federal, state, county, university, and private resource personnel working with companion animals, livestock, and wildlife. Specimens were collected primarily from dogs and cattle, with initial detections of female adult stage ticks by stakeholders associated with parasitology positions (e.g., entomologists and veterinary parasitologists). Initial county tick detections were confirmed with morphological and molecular identifications, and then screened for the presence of animal-associated pathogens (*Anaplasma marginale*, *Babesia* species, *Ehrlichia* species, and *Theileria orientalis*), for which all tests were negative. Herein, we describe the identification and confirmation of these tick specimens as well as other results of the surveillance collaboration.

## 1. Introduction

*Haemaphysalis longicornis* Neumann 1901, the Asian long-horned tick, is an invasive and exotic tick species in North America, an indirect human health threat, and a menace to livestock, companion animals, and wildlife [1]. Since its original detection in the fall of 2017 in New Jersey, the occurrence of *H. longicornis* has been confirmed in 15 states [2]. In its native range, this species can transmit a number of pathogens, uses animals for dispersal, and is an economic threat to the agricultural and livestock industries [1,3]. In geographic areas where *H. longicornis* has invaded, expanded, and proliferated (e.g., New Zealand), exotic and established tick populations can become hyperintense, where thousands of ticks can be found parasitizing a single animal as displayed on a pet sheep in New Jersey [4] and potentially causing tick-infested livestock to become quickly anemic [3,5]. Indirect effects associated with infestations by this species include the transmission of bovine pathogens (*Anaplasma marginale*, *Babesia* spp., and *Theileria orientalis*), dog pathogens (*Babesia* spp. and *Hepatozoon canis*), and human pathogens (e.g., *Borrelia*, *Ehrlichia*, *Rickettsia*, and several viruses) [6]. Recently, investigators in Virginia determined field-collected *H. longicornis* were infected with the *Theileria orientalis* Ikeda genotype and demonstrated that study ticks were competent experimental vectors for the Ikeda strain of the pathogen in local cattle [7,8,9,10].

Unlike most native and typical North American tick species, exotic and invasive *H. longicornis* ticks in the United States (US) have a univoltine life cycle and a parthenogenetic reproductive strategy, which permits rapid population growth because every single unmated female tick can lay hundreds of viable eggs and the resulting adults are all instantly reproductive females [3,11]. This species is a three-host tick (i.e., it uses a different host for each of its larval, nymphal, and adult stages and molts to the next stage off the host in the environment), and it feeds on a variety of potential hosts in the US, including humans, livestock (e.g., cow, sheep, goat, horse, and chicken), companion animals (e.g., dog and cat), and wildlife (e.g., white-tailed deer, raccoon, Virginia opossum, elk, coyote, red and gray fox, eastern cottontail rabbit, groundhog, black bear, Canada goose, great-horned owl, red-tailed hawk, brown booby, gray squirrel, and striped skunk) [2,12,13]. Clearly, its parthenogenetic reproductive strategy, univoltine life cycle, and use of a variety of hosts for dispersal and feeding will make management extremely difficult and emphasize the need for prevention and detection for population management. 

In this context, we developed a collaborative tick-surveillance network for the state of Tennessee, which included academic, government, and other stakeholders, with the primary goals of (a) developing a comprehensive tick-surveillance program to detect this species as soon as possible, (b) using those tick collections to develop educational materials for stakeholders and researchers, and (c) documenting the early and first detection of *H. longicornis* in multiple Tennessee counties. Here we report on our collaborative efforts to identify both established and detected populations of *H. longicornis* in Tennessee (March 2019 to February 2020). Additionally, we report identification as morphological and genetic confirmation and determine the potential presence of animal pathogens within the collected ticks with molecular testing. Before this study, *H. longicornis* had not yet been detected in Tennessee, but it was identified in nearby states of North Carolina, Virginia, and West Virginia, and several researchers predicted that populations will expand across North America into Tennessee and westward into Arkansas [14,15,16]. When our study began (March 2019), known hosts of *H. longicornis* in the US were 17 wild and domestic mammal species and one hawk (a mammalian predator) [1,12]. Additionally, ticks had been collected from vegetation in yards, parks, pastures, and forested areas [12,16].

## 2. Materials and Methods

To develop a tick-surveillance network, our approach was to establish partnerships with Tennessee stakeholders to conduct host-targeted tick surveillance from previously identified companion animals, livestock, and wildlife hosts. To build partnerships, we enhanced our established collaborations with relevant Tennessee state departments (Agriculture, Health, Wildlife Resources). University of Tennessee faculty working with animals, such as those in the Departments of Animal Science, Forestry/Wildlife/Fisheries, in the College of Veterinary Medicine (CVM), and university-owned Research and Education Centers (RECs) were contacted to help with passive surveillance. In order to reach a broader community, we expanded our efforts and contacted animal shelters in the eastern region of Tennessee, as well as the general public through community engagement events (e.g., field days, agricultural days, trade shows). We identify participants recruited from these community engagement events as community scientists (formerly referred to as citizen scientists) because they collected ticks in this highly collaborative project to increase scientific knowledge. Additionally, throughout the project period, we hired and trained 20 undergraduate students in tick detection and had them search animals at livestock markets and hunter-check stations associated with the Tennessee Wildlife Resources Agency (TWRA). 

We provided initial information and collection materials to all collectors to help in the collection process, and instructions on how to collect and submit ticks through demonstrations and videos. Educational products (e.g., infographic and posters) for stakeholders and network collectors were developed and reviewed by the coauthors and then shared with the network team. Print materials were developed in Adobe InDesign and Adobe Illustrator (Adobe Systems, San Jose, CA, USA), while how-to videos were compiled and edited in Adobe Premiere Pro (Adobe Systems), and effects and transitions were created in After Effects (Adobe Systems). These educational materials are available on our website (www.tnticks.org) and through University of Tennessee Extension publications [17,18,19]. We hosted and encouraged collaborators to attend training events, such as in-service trainings for Extension agents, virtual meetings or Hot Topics for those unable to visit campus, and we assisted with a two-day “external parasite” workshop with the U.S. Department of Agriculture, Animal and Plant Health Inspection Service (USDA-APHIS). To encourage recruitment and retention, we provided monthly updates of ranked results from different agencies (e.g., top-contributing agency), participating personnel (e.g., top collectors), and the geographical and temporal data (e.g., top tick-infested region), all shown previously to improve collaborations [20,21].

Groups were asked if they would collect ticks from targeted hosts, including companion animals, domestic livestock, and wildlife. To ease collector efforts, the Medical and Veterinary Entomology laboratory at the University of Tennessee Knoxville (UTK) assembled collection kits, each consisting of fifty 15-mL conical vials, each filled with 10 mL of 70% ethanol. Kits also included UTK Institutional Animal Care and Use Committee (IACUC) paperwork with a consent form containing collection and submission instructions, directions for sample labeling (e.g., county, date, host, at a minimum), worksheets to collect additional host information (e.g., age, breed, coat color, health status, etc.), and extra 70% ethanol. We then distributed collection kits to collaborators (those who agreed to collect) throughout Tennessee, with a focus on the eastern region of the state because of the proximity to established *H. longicornis* populations [21]. We contacted collaborators monthly to reinforce collection and submission efforts. Vials with collected ticks and accompanying worksheets were either picked up by the authors, the project team, or delivered by collaborators to the UTK campus for analysis. Tick collection protocols for the listed collaborators were approved by the UTK IACUC (#2192-0419, #2671-0211, and #0561-0814).

Once submissions arrived at UTK, project team members transferred specimens into new 80% ethanol vials to tentatively separate “suspected *H. longicornis*” and “non-*H. longicornis*” ticks pending preliminary analysis. Collected specimens were subsequently identified to species and life stage by means of specialized *Haemaphysalis* taxonomic materials [22] and general diagnostic keys associated with other tick species in the area [23,24,25,26,27,28]. The first *H. longicornis* collections for each infested county discovered were sent to the USDA-National Veterinary Services Laboratories (NVSL) in Ames, Iowa, for morphological confirmation, with requested additional notification of results to Tennessee Department of Agriculture and Health personnel.

After the NVSL had confirmed tentative identifications and returned the subject specimens to UTK, subsequent molecular diagnostic procedures were performed to genetically confirm identifications of those ticks by amplifying 16S ribosomal DNA (rDNA) and the cytochrome c oxidase 1 (*cox-1*) genes, as previously described for *H. longicornis* confirmation identification [29,30,31]. Additionally, verified specimens of *H. longicornis* were screened for animal-associated pathogens. Specifically, screening included *Anaplasma* and *Ehrlichia* via nested PCR amplification of the *groEL* genes [32], *Babesia* via nested PCR amplification of 18S rRNA [33], and *Anaplasma* and *Theilieria* via multiplex qPCR amplification of major surface protein 5 (*msp5*) and major piroplasm surface protein (*mpsp*), respectively [8]. Briefly, ticks were bisected longitudinally, and half of each specimen was saved as a voucher in a −20 °C freezer at UTK. Total DNA from the remaining half was extracted with the QIAamp 96 DNA QIAcube HT kit (QIAGEN, Hilden, Germany) and eluted in 200 µL AE buffer. All extracted DNA was stored at –20 °C until processed for species confirmation. For the identification of each tick (16S rDNA and *cox-1*) and the initial primary PCR reactions (*groEL* and 18S rRNA), a 20-μL reaction was mixed to include 2 μL eluted DNA, 10 μL of 2× DreamTaq HotStart Green PCR mix (ThermoFisher, Waltham, MA, USA), 0.25 µM each of forward and reverse primers, and 6 μL of nuclease-free water (Supplementary Material 1). The nested PCR for *groEL* and 18S rRNA was a 30-μL reaction of 2 μL primary reaction DNA, 15 μL of 2× DreamTaq HotStart Green PCR mix (ThermoFisher), 0.25 µM each of forward and reverse primers, and 11 μL of nuclease-free water (Supplementary Material 1). One positive control (previously positive tick with targeted DNA) and two negative controls (water and MasterMix without DNA, respectively) were used. For detection of *A. marginale* and *T. orientalis* Ikeda, we used a newly developed multiplex qPCR amplification of *msp5* and *mpsp* [7,8]. If a tick was PCR positive (presence of band in a 1.5% agarose gel: 1 X TAE buffer with ethidium bromide for 2 h at 100 V), then that amplicon was bidirectionally sequenced at Eurofins Genomics (Louisville, KY, USA) using Sanger sequencing. Resulting sequences were cleaned and edited in BioEdit [34] and then compared to sequences deposited in GenBank as previously described [35].

## 3. Results

### 3.1. Network Submissions

**Figure 1:**
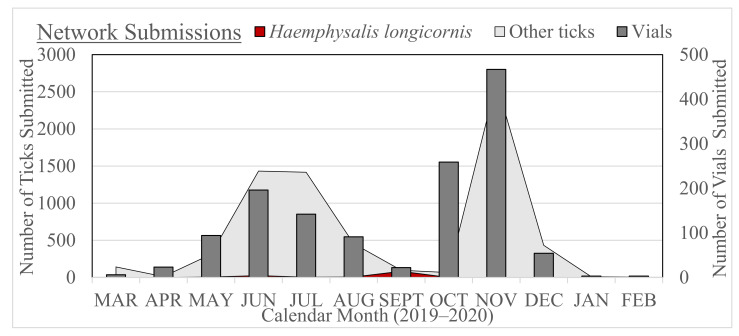
Seasonal distribution of *Haemaphysalis longicornis* (red) and other ticks (light gray) received at University of Tennessee from statewide cooperating network collectors (dark gray), 2019–2020.

A total of 1595 submissions was received from the network during the 12-month period (March 2019–February 2020), yielding 8057 ticks consisting of eight species: 3151 Ixodes scapularis Say, 2246 *Amblyomma americanum* L., 1730 *Dermacentor variabilis* (Say), 400 *Rhipicephalus sanguineus* Latreille, 190 *A. maculatum* Koch, 150 *D. albipictus* (Packard), 144 *H. longicornis*, and 46 *H. leporispalustris* Packard. Sample labeling data for each submitted vial (e.g., county, date, host at a minimum) were nearly complete for all of the submissions, but requested data on the additional worksheets associated with supplemental host information (e.g., age, breed, coat color, health status, etc.) were rarely completed. Only 15% of submissions did not have an associated collection date, 6% did not have an identified host, and 2% did not have an identified county locality. Of the 1595 submissions, only one submission did not have any identifying collection information (county, date, or host). Complete collection data were recorded for 17 of the 22 *H. longicornis* positive submissions, and five submissions with incomplete data were missing only the date of collection. Submissions arrived year-round, with peak times in the summer (May–August) and the fall (October–November), corresponding with the typical temporal activity peak for *A. americanum*, and the traditional white-tailed deer hunting season when many *I. scapularis* were collected, respectively (Figure 1). 

### 3.2. Identification and Confirmation of Haemaphysalis longicornis

**Figure 2:**
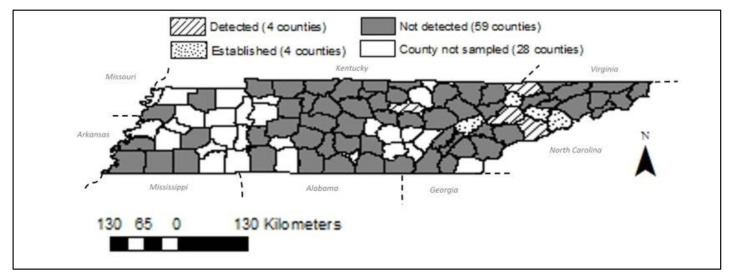
Known occurrence of *Haemaphysalis longicornis* in Tennessee, USA, as determined from samples submitted to the University of Tennessee by a cooperating statewide network of collectors, 2019–2020. We successfully reached 67 counties (gray) of the 95 counties in Tennessee and were able to identify established *Haemaphysalis longicornis* populations in Union, Roane, Jefferson, and Cocke Counties (dotted) and detect populations in Knox, Claiborne, Putnam, and Sevier Counties (striped) within this study period.

Through network partnerships, ticks were collected from 67 counties, and *H. longicornis* were identified in eight of the 95 total counties in Tennessee (Figure 2, Table 1). These eight counties included *H. longicornis* established (six or more specimens were identified) in Union, Roane, Jefferson, and Cocke Counties, and detected (a single life stage of less than six specimens were collected) in Knox, Claiborne, Putnam, and Sevier Counties (Figure 2). Personnel in the network collected and submitted a total of 144 *H. longicornis*, and all three life stages were represented (25 larvae, 69 nymphs, and 50 females). No male *H. longicornis* was collected and submitted in this study.

We deemed the first collection of the tick detected in each Tennessee county as most noteworthy, and each collection was documented by both morphological identification at the USDA-NVSL and molecular sequencing at UTK (Table 2). These 17 ticks were collected from dogs and submitted by animal shelters, collected from cattle by UTK students and staff at livestock markets, or collected from cattle by veterinarians and/or technicians within UTK College of Veterinary Medicine. Of the total 241 dog submissions, 13 (5.4%) vials contained *H. longicornis*, for a mean intensity (previously referred to as burden) of 6.9 *H. longicornis* per infested dog (range: 1–41). Six of 575 sampled cattle (1.04%) were infested with *H. longicornis* and had a mean intensity of 8.5 H. longicornis per infested cow (range: 1–41). We received a single *H. longicornis* from one sampled deer during this study, and this was on a Knox County fawn brought to UTK College of Veterinary Medicine in June 2019. Most of the sampled deer (88.7%) were examined during hunting season (November–December), when *H. longicornis* were either inactive or not collected and submitted. There was one deer found dead at a research and education center in Cumberland County that was highly infested with ticks; only the ears were searched, and a total of 795 *A. americanum* was collected and identified on this animal.

The 17 *H. longicornis* ticks initially collected from eight Tennessee counties (Table 2) were all confirmed morphologically at the NVSL, genetically identical to one another for both the 16S and *cox-1* genes (sequences provided in Supplementary Material 2), and these sequences were also identical to sequences previously amplified from parthenogenetic tick populations from China and submitted to GenBank (16S accession no. MK49888, KX083342, and KP324925; and *cox-1* accession no. MK439888, MF6668880). Importantly, sequences from these ticks were 100% identical to those of ticks collected from vegetation in New Jersey and Virginia, and identified by authors as *cox-1* haplotype 2 (GenBank MT034061). The Tennessee specimens were also PCR negative for the genetic markers associated with *Anaplasma marginale*, *Babesia* spp., *Ehrlichia* spp., and *Theileria orientalis*.

## 4. Discussion

Data presented here provide a snapshot of collections made from March 2019 to February 2020, but the Tennessee tick-surveillance network is still actively collecting ticks. Although tick samples were submitted from every group within the tick collection network, *H. longicornis* ticks were identified from samples collected only by certain network partners. The most successful network collaborators tended to be those with a background in parasitology, including technicians at county rescue and animal shelters, veterinary medicine and laboratory technicians, and students directly involved in the project, as well as community scientists (Table 1). We suspect the varying rates of collection of *H. longicornis* and other ticks may derive from collection efforts limited by competing responsibilities and time available while handling each potential tick-infested animal. Although our study does not present data directly supporting this statement, we speculate that participants who collected many ticks and submitted numerous samples are likely to be those with a greater direct interest in the study [36], and that personnel working at animal shelters and community scientists had a greater interest in removing ticks from animals due to concerns about their animal’s health and welfare. Overall, the efforts of community scientists through passive surveillance, initial detections, and discoveries in our study align well with previously published literature [37,38,39]. 

Our access to animals sold at livestock markets resulted in the identification of *H. longicornis* from several counties because many producers will drive from different counties to sell animals at their preferred market. By searching animals at markets, we were able to collect *H. longicornis* and traceback the tick-infested cattle to owners, confirm *H. longicornis*-infested farms, and prevent those *H. longicornis* from moving onto additional farms. Importantly, through permission and conversation, we then earned trust with those producers who let us collect ticks from their properties to confirm *H. longicornis* populations at those sites and to evaluate the efficacy of available acaricides at killing field-collected *H. longicornis* nymphs in laboratory bioassays [40]. Tick surveillance and subsequent management efforts at livestock markets should be afforded a priority because these locations cohouse animals, allowing for comingling and interchange of animals from multiple farms, both tick-infested and tick-free (e.g., susceptible farm). Previous tick collection work at livestock markets indicated that animals sold at auctions were likely to have the greatest infestation prevalences of uncommon tick species and intensities of common tick species [41]. To our knowledge and according to the livestock market owners we worked with, animal owners do little (if any) pest management before, during, or after livestock sales. Herein, we identify this lack of pest management at livestock markets as a biosecurity threat to animal production.

Previous research identified dogs as excellent sentinels of human cases of tick-borne disease and as a predictor for tick distributions and range expansion [42,43,44,45,46,47,48,49,50]. Initial county detections of *H. longicornis* were largely traceable to animal shelters grooming tick-infested dogs (Table 2); thus, we propose dogs presenting to animal shelters can also help with future invasive-tick-species surveillance. After dogs in Union, Jefferson, and Claiborne Counties were identified as infested, we then observed that other animals (e.g., cattle) were identified in the same counties as infested shortly afterwards (approximately within the next two months). In addition, the *H. longicornis*-infested heifer from Roane County (Table 2) shared an environment with several farm dogs that were also infested with many *H. longicornis*. We recognize here that partnerships with animal shelters are extremely beneficial and should be included with all on-going tick surveillance efforts. Although the exact locations of where these animals are picked up may remain unknown, animal shelters still provide valuable general spatial (county-level) and temporal (date picked up) data for surveillance.

Our morphological and genetic confirmations of the 17 *H. longicornis* identifications are informative and allow for proper archiving of detections. Finding only a single mitochondrial genetic haplotype in Tennessee is notable because local *H. longicornis cox-1* genes previously sequenced in the U.S. comprised at least three different haplotypes [51], and our finding of a single haplotype suggests that these Tennessee ticks are filial clones derived from a single introduction event. Additionally, the identical haplotypes in three disjunct populations (Tennessee, Virginia, and New Jersey) are notable because these haplotypes are common in nearby states with *Theilieria*-infected cattle [7,9,31,51]. Importantly and in hindsight, we should have digitally photographed samples and then deposited those confirmed collection records into a digital repository of physical samples (e.g., before mailing for confirmation, genetic analyses) [52]. Specimens are also accepted at the USDA-NVSL reference collection for permanent preservation. Archiving physical and digital specimens is especially important for new county records and should be standard procedure for future initial tick (and other vector) reports to preserve morphological features.

We collected 144 *H. longicornis* and screened 17 (females and nymphs) of these for evidence of various pathogens, but all of these tests were negative. Although this may seem an important result, the sample size is small, and the negative results do not prove an absence of pathogens in the study area. Conversely, if we had detected a pathogen in any of these samples, it would not prove transmission because all of these ticks were feeding on animal hosts that might have had pathogen-infected blood. Future efforts will be used to screen the rest of these ticks for potential pathogens.

The collection and identification of *H. longicornis* in multiple Tennessee counties highlights the success of this collaborative network in its goals. Remarkably, with a relatively minimal investment ($150,000), and in a short-time period, the statewide network was established by using already existing connections and resources. A majority of funding was used to provide supplies to connections, develop educational material, and hire students to assist with the project. With only a slightly larger investment in tick surveillance, we think that the network can be continued and expanded to include a more informative active-tick surveillance plan (e.g., environmental tick drags in known/unknown sites). Early detection is critical in preventing and controlling arthropod pests and vector-borne diseases in animals and humans. Unfortunately, due to many competing priorities, dedicated resources for this purpose have decreased across numerous states, potentially eroding their ability to quickly and accurately monitor both changes in vector populations and human/animal disease incidence. Nevertheless, based on the success of our passive surveillance efforts, we believe a sustained statewide coordinated network could be established to monitor the presence of ticks and associated zoonotic pathogens of public and animal health significance for a modest annual investment (approximately $200,000/year).

Employment of undergraduate students proved to be a broadly effective strategy because they had blocked hours available to do specific tasks; displayed a variety of tangential interests in the study (e.g., human and/or animal health, disease ecology, ecology, agriculture, science communication); gained an introduction to research and critical thinking (e.g., some students began undergraduate research projects with the network dataset); and some also felt a sense of duty in their work, with noticeable increases in confidence [53,54]. These outcomes mirror a similar experience with undergraduate students in a West Nile virus surveillance project in Montana, which not only provided learning opportunities for students, but also increased vector surveillance capacity [55]. Given that the University of Tennessee system has campuses, Extension offices, and research and education centers across the state, it is possible that vector surveillance can be broadened and expanded to incorporate additional undergraduate learning and internship opportunities.

Enhanced community and stakeholder awareness of *H. longicornis* (and other tick species) was an additional favorable outcome of this project. During the collection period, we were able to work with stakeholders and producers to help in making informed pest management decisions regarding their infested animals and properties.

## 5. Conclusions

Herein we report the results of a highly collaborative statewide tick-surveillance program in Tennessee. Our attempts to be inclusive and incorporate a number of agencies within the project showed us that including personnel trained in parasitology and engaging agencies with a direct connection to animal health and welfare provided the greatest likelihood of substantial tick submissions and of successfully collecting *H. longicornis* ticks, as indicated by higher rates of submissions and tick detections associated with those groups. Through employment of undergraduate students, we could work with producers at livestock markets, and animal shelters submitted many ticks, probably because we provided them with supplies and training and technicians were grooming animals. Additionally, by active engagement with multiple stakeholders, we gained recognition as a trusted resource and were then welcomed onto producer properties for additional collections and studies. Through this study, we detected the first collections of *H. longicornis* in one middle and several eastern Tennessee counties, and we built a cooperative network for statewide passive tick surveillance. These results will be important in monitoring and understanding the further dispersal of *H. longicornis* in Tennessee and in monitoring for incursions by other exotic and invasive tick species.

## Figures and Tables

**Table 1:** Summary of tick collections submitted by cooperating stakeholder groups in Tennessee, USA, March 2019–February 2020.

Network Collector	No. of Vials Submitted	All Tick Statistics (Per Submission)			No. of *Haemaphysalis longicornis* Individuals (No. Vials with *H. longicornis*)
		Mean ± SE	Median (Mode)	Range	
Collections made by Cooperative Stakeholders					
County Animal Shelters (companion animals)	468	6.03 ± 0.787	1 (1)	0–222	70 (11)
Univ. Tennessee Extension Agents	31	1.19 ± 0.097	1 (1)	0–3	0 (0)
Univ. Tennessee Research & Education Centers ^1^	76	2.81 ± 0.346	2 (1)	1–14	0 (0)
Univ. Tennessee Veterinary Medicine	18	3.17 ± 0.793	1.5 (1)	1–13	8 (4)
Tennessee Wildlife Resources Agency (TWRA)	374	8.24 ± 0.673	3 (0)	0–80	0 (0)
Tennessee Department of Agriculture	21	7.10 ± 4.317	3 (1)	1–93	0 (0)
Public (community scientists)	8	3.38 ± 1.523	1.5 (1)	0–13	21 (3)
U.S. Department of Agriculture, Animal & Plant Health Inspection Service–Wildlife Services	36	5.58 ± 0.874	4 (2)	1–19	0 (0)
Undergraduates hired by Univ. Tennessee Medical and Veterinary Entomology laboratory					
Collections from Livestock Markets	492	0.63 ± 0.222	0 (0)	0–90	45 (4)
Collections from Companion Animals	26	3.81 ± 0.872	1.5 (1)	1–19	0 (0)
Wildlife Trapping with TWRA	44	6.02 ± 1.913	2 (1)	0–80	0 (0)
Total	1594	4.56 ± 0.309	1 (0)	0–222	144 (22)

^1^ 1 deer was recovered with 795 ticks on its ears, and all were *Amblyomma americanum*; data were included in text body but not this table.

**Table 2:** Chronological summary of stakeholder submissions of first collection records for *Haemaphysalis longicornis* ticks originating in eight counties of middle and eastern Tennessee, 2019–2020. Bisected specimens are retained as vouchers at the University of Tennessee Medical and Veterinary Entomology laboratory.

County	Date	Host	Number (Life Stage)	Collection Site	USDA-NVSL ^1^ Accession No.
Union	13 May	dog	2 (nymphs)	animal shelter	19-015117
Roane	22 May	cattle (heifer)	5 (females)	Univ. Tennessee Veterinary Medicine	19-015663
Knox	7 June	cattle (bull)	1 (female)	Univ. Tennessee Veterinary Medicine	19-017385
Jefferson	19 July	dog	2 (females)	animal shelter	19-021846
Claiborne	29 July	2 dogs	1 (female) 1 (female)	animal shelter	19-027808 19-027809
Putnam	17 August	dog	3 (females)	animal shelter	19-029860
Cocke	31 August	cattle (heifer)	1 (female)	livestock market	19-027810
Sevier	31 August	cattle (heifer)	1 (nymph)	livestock market	19-027811

^1^ U.S. Department of Agriculture, National Veterinary Services Laboratories.

## Data Availability

Upon publication, data will be made available at our website (www.tnticks.org, accessed on 22 July 2021).

## Supplementary Materials

The following are available online at https://www.mdpi.com/article/10.3390/ijerph18157980/s1, as well as www.tnticks.org. Supplementary Material 1; Table S1: Primers and thermal cycle conditions for amplifying genes used in the molecular identification of *Haemaphysalis longicornis* ticks and potential animal-associated pathogens. Supplementary Material 2; Table S2: Amplified genetic sequences from *Haemaphysalis longicornis* confirming their identification.
