# Supplementary material for: Rapid Discovery and Detection of Haemaphysalis longicornis through the Use of Passive Surveillance and Collaboration: Building a State Tick-Surveillance Network
Source: Int J Environ Res Public Health. 2021 Jul 28;18(15):7980. doi: 10.3390/ijerph18157980 (PMC8345789; doi:10.3390/ijerph18157980)
Supplement: Supplementary file 1 [file ijerph-18-07980-s001.zip › ijerph-1321984-supplementary.pdf]

International Journal of
Environmental Research and Public Health

MDPI

Supplemental 1. Primers and thermal cycle conditions for amplifying genes used in the molecular identification of *Haemaphysalis longicornis* ticks and potential animal-associated pathogens.

Target (Reference)	Gene (band size)	Primers 5′-3′ (10 µM concentration)	Cycling Conditions
Tick DNA [29,30]	16S ribosomal DNA (~455bp)	*16S*_F- 5′ TTA AAT TGC TGT RGT ATT<br>*16S*_R- 5′ CCG GTC TGA ACT CAS AWC	initial denaturation (94°C for 5 min)<br>• 5 cycles of 94°C for 30s, 49°C for 30s, 68°C for 60s;<br>• 5 cycles of 94°C for 30s, 47°C for 30s, and 68°C for 60s;<br>• 5 cycles of 94°C for 30s, 45°C for 30s, and 68°C for 60s;<br>• 25 cycles of 94°C for 30s, 43°C for 30s, and 68°C for 60s;<br>final extension (68°C for 5min)
	cytochrome oxidase I (~820bp)	*COX1*_F- 5′ GGA ACA ATA TAT TTA ATT TTT GG<br>*COX1*_R- 5′ ATC TAT CCC TAC TGT AAA TAT ATG	initial denaturation (95°C for 10 min);<br>• 40 cycles of 95°C for 30s, 55°C for 60s, and 72°C for 60s<br>final extension (72°C for 7 min)
Anaplasma and *Ehrlichia* DNA [32]	*groEL*<br>(~664 bp)	Primary Reaction<br>Gro607F- 5′ GAA GAT GCW GTW GGW TGT ACK GC<br>Gro1294R- 5′ AGM GCT TCW CCT TCW ACR TCY TC	initial denaturation (95°C for 15 min);<br>• 40 cycles of 95°C for 30s, 58°C for 30s, and 72°C for 30s<br>final extension (72°C for 3 min)
	(~315 bp)	Nested Reaction<br>Gro677F- 5′ ATT ACT CAG AGT GCT TCT CAR TG<br>Gro1121R- 5′ TGC ATA CCR TCA GTY TTT TCA AC	initial denaturation (95°C for 15 min);<br>• 35 cycles of 95°C for 30 s, 58°C for 30 s, and 72°C for 30 s<br>final extension (72°C for 3 min)
Babesia DNA [33]	(~1600 bp)	Primary Reaction<br>*Bab18s*_1F 5′- AAG CCA TGC ATG TCT AAG TAT AAG CTT TT<br>*Bab18s*_1R 5′- CTT CTC CTT CCT TTA AGT GAT AAG GTT CAC	initial denaturation (94°C for 10 min);<br>• 35 cycles of 94°C for 30 s, 50°C for 30 s, and 72°C for 60 s<br>final extension (72°C for 5 min)
	(~390 bp)	Nested Reaction<br>*Bab18S2*_437-461.F 5′- AAT CCT GAC ACA GGG AGG TAG TGA C -3′<br>*Bab18S2*_898-873.R 5′- CTA AGA ATT TCA CCT CTG ACA GT -3′	initial denaturation (94°C for 10 min);<br>• 35 cycles of 94°C for 30s, 65°C for 30s, and 72°C for 30s<br>final extension (72°C for 5 min)
Anaplasma marginale and *Theileria orientalis* DNA [8]	qPCR	*Theileria orientalis*<br>Forward primer 5′ –<br>GCA AAC AAG GAT TTG CAC GC<br>Reverse primer 5′ –<br>TGT GAG ACT CAA TGC GCC TAG A<br>Universal probe 5′ –<br>NED-TCG ACA AGT TCT CAC CAC MGB-NFQ<br>*Anaplasma marginale*<br>Forward primer 5′ –<br>TTG GCA AGG CAG CAG CTT	Initial denature (95°C for 10 min);<br>45 cycles of 95°C for 15s, 60°C for 60s

	Reverse primer 5′ –<br>TTC CGC GAG CAT GTG CAT

Supplement 2. Amplified genetic sequences from *Haemaphysalis longicornis* ticks, confirming their identification.

>16S sequences for all of the Tennessee ticks were 100% identical to *H. longicornis* GenBank sequences (MK439888, KX083342, KX450293, KU986714, KP324925, KJ710084, AB819205, JX051073, JX051071, JX051070, JX051069, JX051066, JX051064, MW602986, JF979373, KR259991)

CTAAGAGAATGGATTTTCAAAAAAATTCTTTTTTAAGTTTAAAAATTAAAGTTATTTTTATTTGTGAAGAAAC
AATAATAAAAATTAAAGACAAGAAGACCCTATGAATTTTTATAAACTTTAATATTTAATTAAATATTAAAG
TTTATTTAATTGGGGCGATTGAGAAAGATAAAAAACTTTTTTTTTATTTAAAGAGATCCATTATTAATGATTT
TATGTAAAAAATACTCTAGGGATAACAGCGTAATAATTTTAGATAGATCTTATAGAAAAAATAGTTTGCGA
CCTCGATGTTGGATTAGGATACTTGTTTAATGAAGAAGTtAAATAAAGAAG

>COX-1 sequences for all of the Tennessee ticks were 100% identical to *H. longicornis* GenBank sequenecs (MK439888, MF6668880)

TAAGAATTTTAATTCGAATAGAACTAGGGCAACCTGGTACATTAATTGGAAATGATCAAATCTATAATGTA
ATCGTTACCGCTCATGCTTTTATCATAATTTTTTTTATAGTTATACCAATTATAATTGGAGGATTTGGAAATTG
ATTAGTGCCATTAATATTAGGCGCTCCTGACATAGCATTTCCTCGAATAAATAACATAAGATTCTGACTATT
ACCTCCCTCTATATTCTTATTAATTAATTCATCTTTAGTTGAAAGAGGGGCAGGTACAGGATGAACTGTATA
TCCTCCATTATCATCTAATTTGTCCCATTATGGACCCTCAGTAGATATAGCAATTTTTTCACTCCACTTAGCA
GGAGCCTCATCAATTTTAGGAGCTATTAATTTCATTACAACTATCATTAATATACGATCTTTAGGAATAACTT
TAGAACGAATACCGCTATTTGTTTGATCAGTTCTAATTACCGCAATTCTCCTTCTGCTATCCTTACCTGTTCTT
GCCGGTGCAATTACCATACTCTTGACAGATCGAAATTTTAACACCTCATTCTTTGACCCCTCAGGAGGAGGA
GACCCAATTTTATATCAACACTTATTTTGATTTTTTGGACACCCAGAAGTTTATATTTTAATTTTACCAGGATT
TGGAATAATTTCACAAATCATTTGTTTTCATACAGGTAAAAAAGAACCTTTTGGTAATTTAGGCATAA-
TTTATGCTATATTAGCAATTG
